# Long Noncoding RNA and Circular RNA: Two Rising Stars in Regulating Epithelial-Mesenchymal Transition of Pancreatic Cancer

**DOI:** 10.3389/fonc.2022.910678

**Published:** 2022-06-03

**Authors:** Xiaoying Yang, Cheng Qin, Bangbo Zhao, Tianhao Li, Yuanyang Wang, Zeru Li, Tianyu Li, Weibin Wang

**Affiliations:** Department of General Surgery, State Key Laboratory of Complex Severe and Rare Diseases, Peking Union Medical College Hospital, Chinese Academy of Medical Sciences and Peking Union Medical College, Beijing, China

**Keywords:** long noncoding RNA, circular RNA, epithelial-mesenchymal transition, pancreatic cancer, competing endogenous RNA

## Abstract

Pancreatic ductal adenocarcinoma (PDAC) is a highly malignant tumor with especially poor prognosis. However, the molecular mechanisms of pancreatic oncogenesis and malignant progression are not fully elucidated. Epithelial-mesenchymal transition (EMT) process is important to drive pancreatic carcinogenesis. Recently, long noncoding RNAs (lncRNAs) and circular RNAs(circRNAs) have been characterized to participate in EMT in PDAC, which can affect the migration and invasion of tumor cells by playing important roles in epigenetic processes, transcription, and post-transcriptional regulation. LncRNAs can act as competing endogenous RNAs (ceRNA) to sequester target microRNAs(miRNAs), bind to the genes which localize physically nearby, and directly interact with EMT-related proteins. Currently known circRNAs mostly regulate the EMT process in PDAC also by acting as a miRNA sponge, directly affecting the protein degradation process. Therefore, exploring the functions of lncRNAs and circRNAs in EMT during pancreatic cancer might help pancreatic cancer treatments.

## Introduction

Pancreatic ductal adenocarcinoma (PDAC) is one of the malignant tumors with poor prognosis, and currently, the only possible cure is through surgical removal. The relatively low overall survival (11%) is caused by the deep-seated position of the pancreas, the lack of appropriate diagnostic approaches, difficulties in performing a tissue biopsy, and its low response rate to radiotherapy or chemotherapy (1). Most pancreatic cancer patients are diagnosed at an advanced stage with metastasis, and therefore surgical management is unavailable for over 80% of patients (2). Metastatic pancreatic cancer has poor prognosis with a survival rate of 5% at 5 years (3, 4). For pancreatic cancer patients undergoing surgery, 5-year survival of them is also lower than 30%, which is critically ascribed to the distance metastases after surgery (5). Therefore, understanding the mechanism of PDAC metastasis is pivotal for the development of prognostic biomarkers and therapeutic targets.

Epithelial-mesenchymal transition (EMT) is a critical pathophysiological step that associates PDAC cells with invasion, migration, and acquisition of stem cell-like phenotype (6). During the process of EMT, PDAC cells change the protein expression with the loss of epithelial markers such as E-cadherin, cytokeratins, claudin, and occludin, and the elevation of mesenchymal markers such as N-cadherin, vimentin, and fibronectin (7). These changes lead to the loss of cellular polarity, disruption of normal cell adhesion, remodeling of the cytoskeleton, and changes in cell apoptosis, which collectively translate into enhanced invasion, migration, and metastasis (8). The mechanism behind this process is complex and involves different EMT-inducible transcription factors, such as SNAIL, SLUG, ZEB1, ZEB2, and Twist (9). Although the entire process of EMT is not yet completely understood, recent studies have shown that a variety of non-coding RNAs (ncRNAs), which include circRNA and lncRNAs are involved in regulating the EMT process of pancreatic cancer cells (10–14).

Long noncoding RNAs (lncRNAs) are operationally defined as RNA transcripts longer than 200 nucleotides, and current evidence showed that they could not encode peptides, since their locations most in the nucleus with low expression levels and little primary sequence conservation. These molecules serve vital roles in gene regulation, including modulating gene activating and silencing, alternative splicing, post-translational regulating, and chromosome inactivation, and perform these functions through different pathways to contribute not only to the development and metabolism but also to the cancer progression (15). Circular RNAs (circRNAs) have emerged as a large category of non-coding RNA molecules, many of which are highly abundant, conserved, and play important physiological and pathophysiological roles. Besides, these molecules also have exceptional promise as diagnostic, prognostic, and predictive biomarkers (16). CircRNA and lncRNAs not only regulate the intracellular EMT pathway but also can be encapsulated by extracellular vesicles (EVs), such as exosomes, thus regulating the whole EMT pathway of multiple cells in the microenvironment of pancreatic cancer (17, 18).

In this review, we focused on recent literature findings and review the mechanism of circRNA and lncRNA involved in the regulation of EMT in pancreatic cancer.

## EMT in Pancreatic Cancer

**Figure 1:**
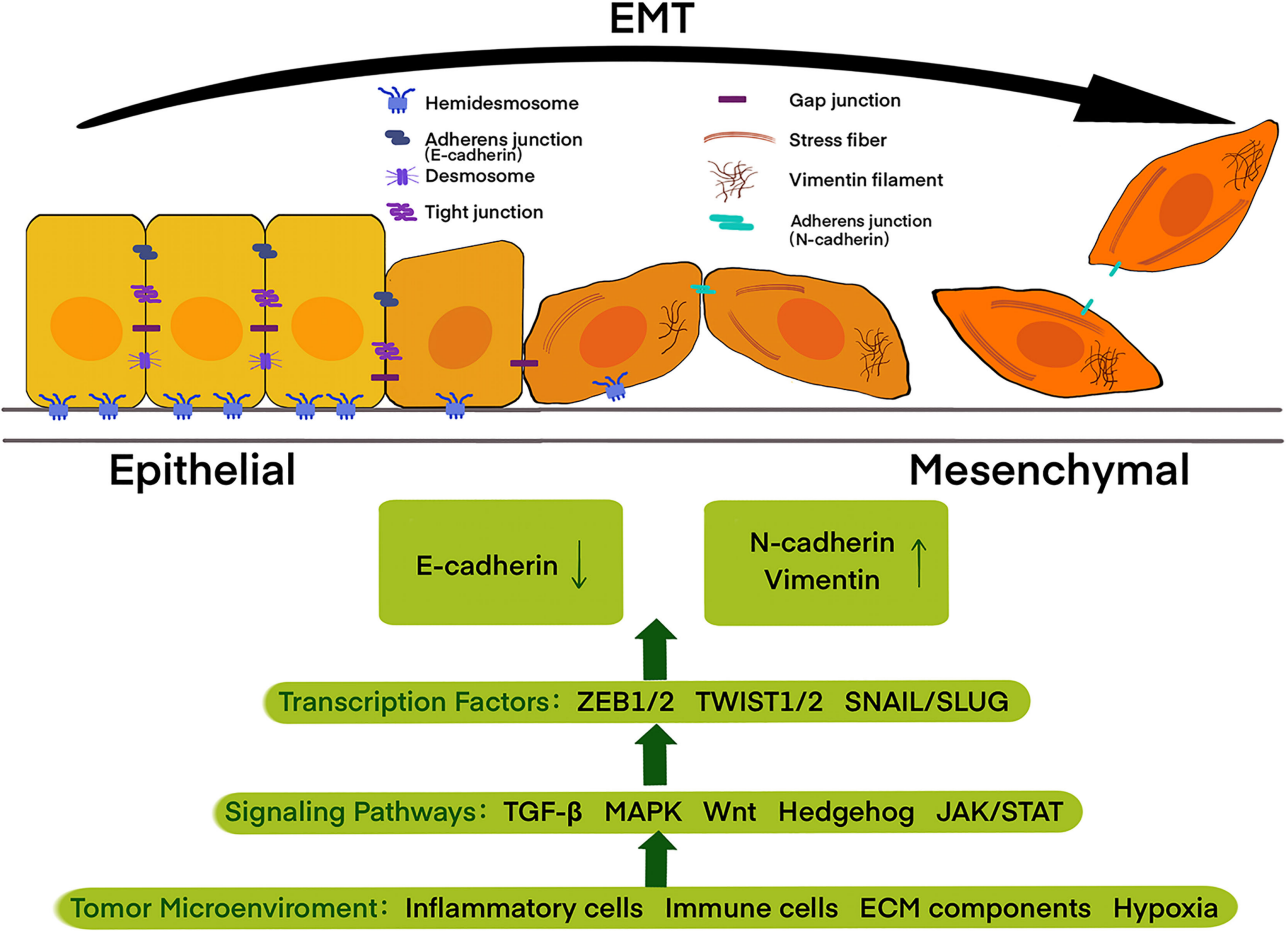
Epithelial cells with apical-basal polarity are tethered to the basement membrane by hemidesmosomes and held together by adherens junctions, tight junctions, and desmosomes, while mesenchymal cells display an extensively reorganized cytoskeleton with front-to-back polarity. Cells during epithelial-mesenchymal transition (EMT) undergo a left to right transition, with the loss of epithelial features accompanied by the acquisition of mesenchymal features. Tumor microenvironment including inflammatory cells, immune cells, ECM components, and also physical constraints, such as hypoxia, trigger signaling pathways, including the TGF-β, MAPK, Wnt, Hedgehog, and JAK/STAT, thus activating EMT-promoting transcription factors of the TWIST, SNAIL and ZEB families and further regulate the expression of E-cadherin, N-cadherin, and vimentin.

EMT is a dynamic and reversible process through which epithelial cells present a mesenchymal-like phenotype, which is defined by changes in cell surface markers, cell morphology, and migration functions (19). It is now widely accepted that EMT is associated with invasion, dissemination, metastasis, as well as a poor diagnosis in epithelial cancers, including pancreatic cancer (20, 21). In the past few decades, besides the multi-faceted consideration of EMT-related invasiveness, the switch of cell surface markers from E-cadherin to N-cadherin and vimentin has been increasingly used to monitor the EMT process during cancer development as the hallmarks (22).The process of EMT contains the decomposition of epithelial cell-cell contacts, the loss of cell polarity, and the acquisition of mesenchymal features (**Figure 1**).

E- and N-cadherin both belong to type-I classical cadherins with similar sizes of 120 kDa and130 kDa. but different structures. Cadherin cytoplasmic tails attach with β-catenin which connects to α-catenin, thus forming the cadherin-catenin adhesion complex (23). E-cadherin loss is described as a key event of EMT, leading to decreased cell-cell adhesion, permitting the separation of individual cells from the primary tumor mass, and therefore represents invasion and metastasis in early tumor progression. In contrast with E-cadherin, N-cadherin is prevalent in non-epithelial tissues and is expressed in different types of cells such as neural cells, endothelial cells, stromal cells, and osteoblasts (24). N-cadherin is upregulated while E-cadherin is downregulated during EMT in cancers and this “cadherin switch”, which is regulated by several factors and pathways, is associated with enhanced migratory and invasive traits, which caused inferior patient survival rate (25). Vimentin is a type III intermediate filament (IF) protein, which supports other cellular organelles in EMT due to its viscoelastic attributes and directs cell migration through forming cellular protrusions and increasing the migratory ability of cells (26).

The EMT process is orchestrated by a suite of transcription factors (such as TWIST, SNAIL, SLUG, and ZEB1) (27). SNAIL1 (also known as SNAIL) and SNAIL2 (also known as SLUG) can activate the EMT programming during development and cancer. They repress epithelial genes expression by binding to E-box sequences in the proximal promoter region of the E-cadherin gene (28, 29). TWIST expression not only down-regulates E-cadherin expression as SNAIL does, but also induces N-cadherin expression. TWIST1 and TWIST2 form homodimers and also heterodimers, regulate E-box DNA binding and transcription regulation through mediating histone mono-methylation on different positions of E-cadherin and N-cadherin promoters (30, 31). Zinc finger E-box binding homeobox 1 (ZEB1) (also known as δEF1) and ZEB2 (also known as SMAD interacting protein 1 (SIP1)), members of the ZEB family, are characterized by two separated highly conserved clusters of zinc fingers. ZEB1 and ZEB2 have been found to bind CACCT sequences, including E2 boxes of the E-cadherin promoter, by their zinc finger clusters. Thus they can repress the transcription activity of the E-cadherin promoter (32).

EMT is also controlled by a complicated network of signaling pathways, including the transforming growth factor-β (TGF-β) signaling pathway, the mitogen-activated protein kinase (MAPK) signaling pathway, the Wnt signaling pathway, the hedgehog (HH) signaling pathway, the Hippo-Yes-associated protein (YAP) signaling pathway, the Janus kinase (JAK)/signal transducer and activator of transcription (STAT) signaling pathway, etc (33). Type 1 TGF-β receptor (TβR1) needs be phosphorylating by type 2 TGF-β receptor and activated by TGF-β signals thus initiating classical TGF-β/Smad signaling. This signaling continues to phosphorylate Smad2 and Smad3, which activates the transcriptions of SNAI1 and SLUG by binding directly to their promoters (34, 35). TGF-β can also activate ERK, Jun N-terminal kinase (JNK), MAPK through the β-catenin/T-cell factor (TCF) pathway (36). MAPK signaling in turn activates the TGF-β signaling pathway, probably because of the co-stimulation, leading to repression decreasing in E-cadherin and increasing in N-cadherin and vimentin (37). In addition, MAPK can stabilize SNAIL1 and increase its activity by inhibiting glycogen synthase kinase-3β (GSK-3β), which can phosphorylate SNAIL and induce SNAIL inactivation and degradation (38, 39). In canonical Wnt signaling, Wnt ligands bind and inhibit GSK3β, hence preventing β-catenin and SNAIL phosphorylation, ubiquitylation and degradation thus promoting EMT (40). In HH signaling, the ligand binds to patched (PTC) receptors to activate glioma-associated oncogene (GLI) family transcription factors (41). SHH/GLI1 expression induced upregulation of expression of S100A4, a member of the S100 gene family, which was found to promote the expression of TWIST and SNAIL during EMT but with an unclear mechanism (42). The Hippo-YAP was found to increase the expression of ZEB2 by binding to the promoter of ZEB2 (43). In JAK/STAT pathway, STAT1 is known to involve in anti-tumor immunity, while STAT3 is found to act as a transcriptional activator through binding to the promoter of LIV-1 genes in a tyrosine phosphorylation-dependent manner, resulting in the overexpression of LIV-1, which can stabilize Snail by inactivating of GSK3β, leading to EMT progress (44).

EMT is also regulated by various pro-invasion signals of the tumor microenvironment (TME), which is defined as a cellular and physical environment surrounding the tumor cells, including inflammatory cells, immune cells, ECM components, and so on (45). During tumor programming, tumor cells recruit fibroblasts and immune cells which in turn secrete cytokines to impact tumor development and have been discovered to directly regulate the EMT process. TGF-β, secreted by tumor-associated platelets, fibroblasts, and tumor cells, can induce SNAIL1 and SNAIL2 *via* SMAD signaling in PDAC cells (46). Tumor necrosis factor-α (TNFα) is shown to activate NFκB signaling pathway thus inducing the expression of EMT transcription factors, including TWIST1, SNAIL2, and ZEB1/2 (47–49). Interleukins released by endothelial cells, immune cells, and fibroblasts can also contribute to EMT. Interleukin 6 (IL-6) increased TWIST1 and SNAIL1 expressions and promotes EMT (50). Several extracellular matrix (ECM) proteins, including Fibronectin, Collagen-I, and Hyaluronan are also implicated in controlling EMT. Fibronectin can induce SNAIL1 expression partially through binding to integrin receptors, and can also cooperate with TGFβ to activate the downstream ERK/MAPK kinases thus inducing EMT (51). Collagen-I can stabilize SNAIL1 by activating downstream SRC/ERK2 to promote tumor cell EMT (52). Hyaluronan can bind to tumor cells and activate LOX expression, which is a copper-dependent amine oxidase that catalyzes the cross-linking of collagens and elastins in the ECM, in turn, upregulates TWIST1 expression to promote EMT (52).

Hypoxia condition is another element affecting EMT, which is found to be related to the ability of tumor invasion and metastasis and increases tumor glycolysis and angiogenesis by inducing relevant gene expressions through hypoxia-inducible factors (HIFs) (53). Hypoxia-inducible factor-1α (HIF-1α) can increase SNAIL1 protein stability, resulting in the suppression of E-cadherin (54). Besides, HIF-1α directly binds to the TWIST1 promoter to induce TWIST1 expression (55). In addition, together with TGF-β, HIF1α can promote SNAIL1 nuclear translocation and induce EMT (56).

EMT process has been involved in not only invasion and migration but also resistance to chemotherapy and radiotherapy. It was proved that cells undergoing EMT increased chemoresistance and radioresistance by acquiring stem-like properties, enhancing survival pathways, preventing apoptosis, and activating signaling pathways involved in DNA damage repair and cell cycle progression (57–60).

Mesenchymal-like tumor cells produced by EMT have shown the characteristics of self-renewal, radiation resistance, and drug resistance of tumor stem cells. Therefore, the study of EMT will not only help to deepen the understanding of tumor progression, but also help tumor treatment.

## lncRNA

**Figure 2:**
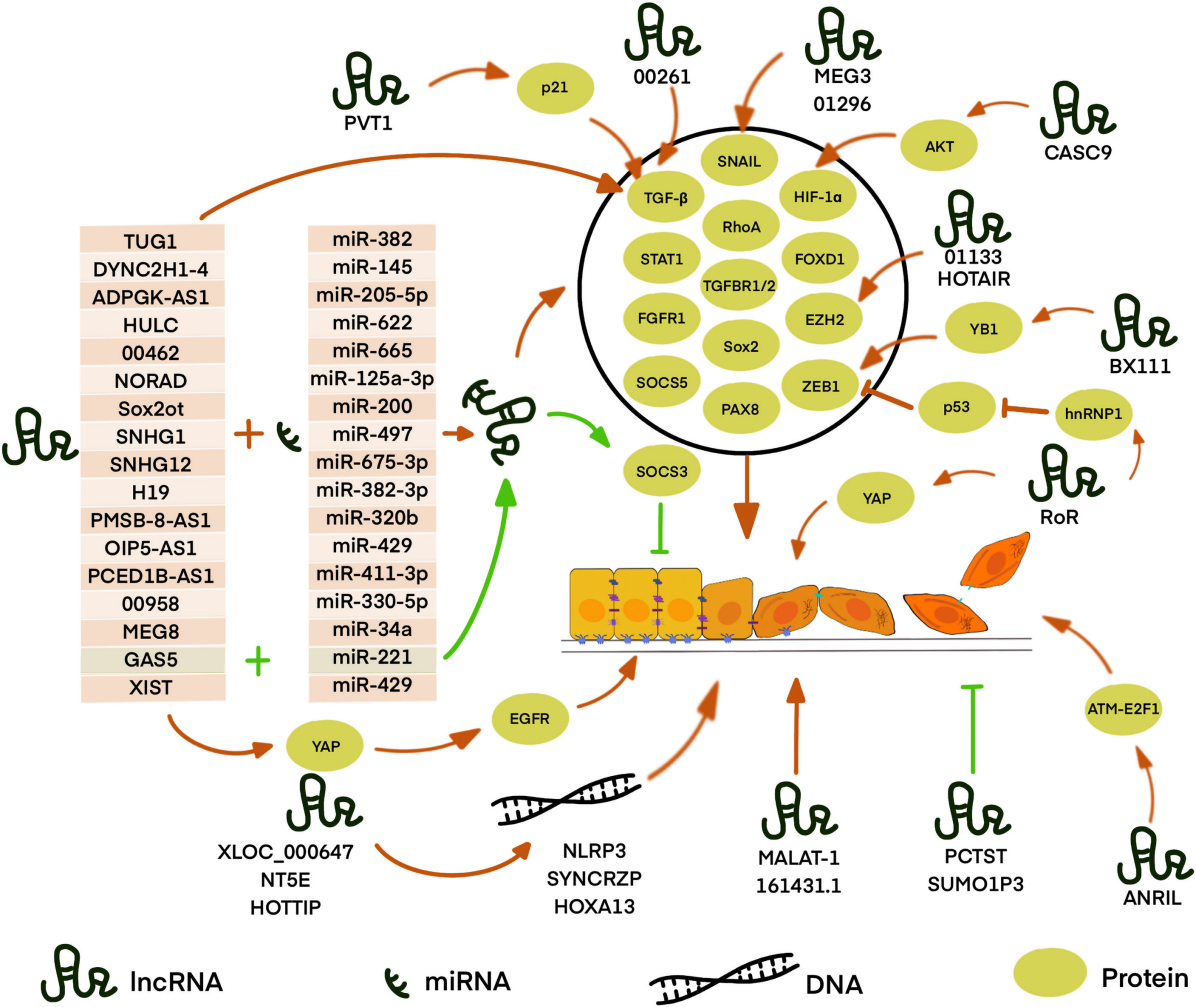
LncRNA is involved in EMT in PDAC through different pathways. Most known LncRNA can act as competing endogenous RNAs (ceRNA) to sequester target miRNA away from mRNA or DNA thus regulating the expression of downstream proteins. LncRNAs, such as XLOC_000647, NE5T, and HOTTIP, can regulate gene expression by binding with local chromatin architectures which localize physically nearby. LncRNAs,such as RoR, BX111, LINC01133, and CASC9, can directly regulate or interact with EMT-related proteins or further affect the expression of its downstream targets. However, some lncRNAs, such as MALAT-1, TUG1, LINC00261, PVT I, ANRIL, MEG3, HOTAIR, SUMO1P3, and LINC01296, play an important role in the EMT process with unclear mechanisms. Red arrows: the pathway promotes EMT, green arrows: the pathway inhibits EMT, the black circle: the proteins regulated by ceRNA and positively regulates EMT.

lncRNAs are RNA transcripts longer than 200nt without protein-coding potential. They are recognized to govern substantial biological processes including cell growth, differentiation, and proliferation (61–63). Accumulated evidence demonstrated that lncRNAs are aberrantly expressed in different cancers (64, 65). Some of them are also involved in oncogenic or tumor-suppressive pathways and have diagnostic and prognostic values (66, 67). Recent studies have confirmed the importance of lncRNAs in tumor growth and metastasis, including lncRNA-XIST in gastric cancer, lncRNA-MALAT1 in colorectal cancer, and lncRNA-PVT1 in gastric cancer, etc (68–70). LncRNAs can regulate gene expressions in different ways, including chromosome remodeling, transcription, as well as post-transcriptional modification (71, 72). Nowadays, increasing data suggests that lncRNAs play important roles in the EMT progression of PDAC (**Figure 2**). The expression change in PDAC, molecular mechanisms, and related genes of these lncRNAs are summarized in **Table 1**.

**Table 1:** LncRNAs in PDAC development through EMT regulation.

LncRNA	Expression change in PDAC	Relevant target genes	Known molecular mechanisms	Reference
TUG1	Up	EZH2, SMAD1/2	CeRNA	(73, 74)
DYNC2H1-4	Up	ZEB1	CeRNA	(75)
ADPGK-AS1	Up	ZEB1	CeRNA	(76)
LINC00462	Up	TGFBR1/2	CeRNA	(77)
NORAD	Up	RhoA	CeRNA	(78)
Sox2ot	Up	Sox2	CeRNA	(79)
SNHG1	Up	FGFR1	CeRNA	(80)
H19	Up	SOCS5, STAT3	CeRNA	(81)
PMSB-8-AS1	Up	STAT1	CeRNA	(82)
OIP5-AS1	Up	FOXD1	CeRNA	(83)
PCED1B-AS1	Up	HIF−1α	CeRNA	(84)
LINC00958	Up	PAX8	CeRNA	(85)
MEG8	Up	SNAIL	CeRNA	(86)
GAS5	Down	SOCS3	CeRNA	(87)
XIST	Up	ZEB1,YAP	CeRNA	(88)
SNHG12	Up	miR-320b	CeRNA	(89)
RoR	Up	ZEB1	p53-ZEB pathway, Hippo/YAP pathway	(90, 91)
BX111	Up	HIF-1α,	Hypoxia-induced EMT	(92)
LINC01133	Up	EZH2	Wnt/β-catenin signaling	(93)
XLOC_000647	Up	NLRP3	Local regulators of its nearby gene	(94)
NT5E	Up	SYNCRIP	Local regulators of its nearby gene	(95)
HOTTIP	Up	HOXA13	Local regulators of its nearby gene	(96)
MALAT-1	Up	Sox2	Unknown	(97)
LINC00261	Up	E-cadherin	Unknown	(98)
PVT1	Up	P21	Unknown	(99, 100)
ANRIL	Up	ATM-E2F1	Unknown	(101)
MEG3	Up	SNAIL	Unknown	(102)
HOTAIR	Up	EZH2	Unknown	(103)
LINC01296	Up	SNAIL	Unknown	(104)
AL161431.1	Up	unknown	Unknown	(105)
PCTST	Down	E-cadherin and repressed vimentin	Unknown	(106)
SUMO1P3	Down	vimentin	Unknown	(107)

### LncRNA: Binding miRNA as Competing Endogenous RNAs (ceRNA)

One of the most important functions of lncRNA is to compete for the miRNA binding site through partial complementarity as a ceRNA.

When lncRNA act as ceRNA, they can interfere with the process of EMT by affecting transcription factors. Gao et al. investigated that lnc-DYNC2H1-4, as a sponge of miR-145, upregulates ZEB1, which leads to upregulation and downregulation of vimentin and E-cadherin, respectively (75). Due to the discovery of the direct interaction between miR-145 and ZEB1 in many solid cancers, it was suggested that lnc-DYNC2H1-4 participates in EMT by the miR-145/ZEB1 pathway (108–110). Moreover, *in vitro* and *in vivo* investigations by Song et al. indicated that lncRNA ADPGK-AS1 inhibits miR-205-5p, which could directly target ZEB1, and therefore induces EMT (76). Also, Shen et al. investigated that lncRNA X-inactive specific transcript (XIST) promotes PDAC cell EMT by acting as a sponge for miR-429, which can directly bind to ZEB1 mRNA, to regulate ZEB1 expression (88). Besides, Zou et al. reported that XIST promotes the expression of YAP, leading to TGF-β1-induced EMT *via* up-regulation of EGFR (111). Li et al. figured out a similar mechanism that lnc-Sox2ot regulates the expression of Sox2 by binding to the miR-200 family and therefore promote EMT in PDAC, thus produce an effect on TNM stage and overall survival rate of PDAC patients (79). Sox, which contributes to the metastasis in various tumor tissue, has been reported to promote EMT *via* regulation of the WNT/β-catenin signal (112). L. Wu et al. revealed that lncRNA OIP5-AS1 can sponge miR-429, which targets FOXD1, thereby activating the ERK pathway and EMT in PDAC (83). S. Chen et al. used LINC00958 as a ceRNA to competitively sponge miR-330-5p and regulate paired box 8 (PAX8), and then promote the EMT process in PDAC. However, further studies are required to fully understand the mechanisms of the miR-330-5p/PAX8 axis on the regulation of EMT in PDAC cells (85). Terashima et al. elucidated that lncRNA maternally expressed gene 8 (MEG8) reduces the expression of miRNA-34a gene, thus up-regulated the expression of SNAIL transcription factors, which repressed the expression of E-cadherin and induced EMT (86). However, there are few reports on the mechanism of the interaction between miRNA 34a and SNAIL. A previous study suggested that STAT1 and STAT3 have opposing roles in the tumor process. STAT1 signaling acts as a tumor suppressor by inhibiting angiogenesis, tumor growth, metastasis, and promoting apoptosis. Alternatively, the STAT3 pathway is implicated in oncogenic progression (113). However, H. Zhang et al. demonstrated that lncRNA PMSB8-AS1 promotes EMT progression in PDAC *via* upregulation of STAT1 by sponging miR-382-3p. They found that STAT1 can transcribe PD-L1, which mediates the inactivation of T cells and promotes invasion and migration, thus participate in EMT process (82). In addition to the transcription factors, some transcription regulatory proteins can also be affected by ceRNA, which in turn affects EMT process. In L. Zhao’s study, lncRNA taurine upregulated gene 1 (TUG1) acts as a molecular sponge for miR-382 and regulates its target, enhancer of zeste homolog 2 (EZH2), which could contribute to the cell proliferation, migration and EMT formation in PDAC (73). A previous study has reported that EZH2 inhibits tumor suppressor genes such as … *via* trimethylation of H3K27 (114). However, the detailed mechanism of EZH2-regulated EMT needs further study. In addition, some cellular receptors and molecular pathways are also affected by the lncRNA acted as ceRNA. B. Zhou et al. demonstrated that the overexpression of linc00462, which is a target of miR-665, enhancing the expression of TGFBR1 and TGFBR2, thus activating the TGF-β/SMAD2/3 pathway and accelerating EMT processing in PDAC cells (77). Moreover, Li et al. reported that lncRNA NORAD act as a ceRNA to regulate the expression of the small GTP binding protein RhoA by sequestering its inhibitor, miR-125a-3p, thereby promoting hypoxia and EMT (78). RhoA is a member of the Rho GTPase family, which was reported to facilitate the EMT process probably by promoting actin cytoskeleton reorganization, leading to the promotion of cell attachment and motility (115). Besides, S. Chen et al. found that lncRNA small nucleolar RNA host gene 1 (SNHG1) competes for the miR-497 binding site as a ceRNA, thus regulating FGFR1 expression to promote the EMT process, shown by changes in the expression levels of E-cadherin, N-cadherin, and vimentin. The detailed mechanisms should be investigated to fully clarify the contributions of the SNHG1-miR-497-FGFR1 interaction in the activation of EMT in future studies (80). Wang et al. discovered that the lncRNA H19/miR-675-3p signaling axis plays an important role in maintaining the EMT process and promoting cancer cell proliferation of PDAC cells, which possibly by directly targeting suppressor of cytokine signaling 5 (SOCS5) and thus activating the STAT3 pathway (81). SOCS3 is another member of the SOCS family that plays a significant role in EMT progression (116). Liu et al. showed that overexpression of lncRNA growth arrest-specific 5 (GAS5) suppresses the proliferation, migration, gemcitabine resistance, stem cell-like properties, and EMT of PDAC cells by directly binding to and suppressing miR-221 and enhancing SOCS3 expression, which can inhibit the EMT and tumor stem cell accumulation. They suggested that lncGAS5 acts as a tumor suppressor (87). Previous studies reported that overexpression of SOCS3 suppresses JAK2/STAT3-signaling activation thus inhibiting the JAK2/STAT3-mediated EMT in breast cancer (117, 118). Thus there is supposed to be the same pathway in PDAC. In Y. Zhang et al.’s study, lncRNA PC−esterase domain containing 1B−antisense RNA 1 (PCED1B−AS1) was shown to target miR−411−3p, resulting in upregulation of HIF−1α, thus participating in promoting proliferation, invasion, and EMT in PDAC (84). Some lncRNAs as ceRNAs can mediate EMT by playing other roles.Cao et al. revealed that lncRNA SNHG12 promotes PDAC cells invasion and EMT by absorbing miR-320b, which was affirmed as a pivotal element in other cancers that suppressed proliferation, migration, invasion, and EMT. Thus in-depth studies are needed to further indicate the related other mechanisms of the miR-320b/EMT axis (89). In general, lncRNA can act as ceRNA to regulate EMT process through various signaling pathways and downstream molecules.

### LncRNAs Function as Stabilizating, Regulating, Remodeling DNA, RNA, and Protein

LncRNAs also function in chromatin and genomic structural remodeling, RNA stabilization, transcriptional regulation, and protein stability (119, 120).

Zhan et al. found that lncRNA regulator of reprogramming (RoR) increases levels of mesenchymal markers N-cadherin, and decreases levels of epithelial markers E-cadherin and induces EMT, and EMT-associated cell proliferation, invasion, and tumourigenicity in PC probably by ZEB1 pathway with the mechanism still unclear (90). A previous study showed that lnc-RoR can suppress p53 translation through direct interaction with heterogeneous nuclear ribonucleoprotein I (hnRNP I), which can bind to p53 mRNA to stimulate p53 translation (121). P53 was found to transactivate the miR-200c and miR-192, which can bind to ZEB1/2 mRNA, thus repressing ZEB1/2 translation (122). Therefore, they suggested that lnc-ROR repressed EMT in PC probably by inhibiting p53-ZEB pathway. Besides, in endometrial cancer, p53 was found to promote the expression of ZEB1 by directly binding to the promoter of miR-130b, the negative regulator of ZEB1, and inhibiting its transcription (123). Besides, W. Chen et al.’s study demonstrated that lnc-RoR overexpression activates the Hippo/YAP pathway and then promotes EMT (91). However, the mechanism by which lnc‐RoR affects YAP needs further exploration. Additionally, Deng et al. found that lncRNA BX111 recruited transcriptional factor Y-box protein (YB1) to its promoter region thus activating the transcription of ZEB1. They also revealed that BX111 transcription is induced by HIF-1α, which contributed to the hypoxia-induced EMT (92). Moreover, Y. Liu et al. evaluated that LINC01133 can promote EMT and metastasis in PDAC by interacting with EZH2 and epigenetically regulating axis inhibition protein 2 (AXIN2), directly activating β-catenin, thus influencing Wnt/β-catenin signaling (93). Hu’s results indicated that overexpression of lncRNA XLOC_000647 results in down-regulating the expression of NOD-like receptor family pyrin domain-containing 3(NLRP3) and inhibition of EMT-induced cell invasion (94). lncRNA XLOC_000647 might act as local regulators of its nearby gene, NLRP3, which was located at 25 kb of the XLOC_000647 downstream. Recent studies have validated that NLRP3 can activate inflammatory cytokines and promotes the metastasis of melanoma cells and hepatocellular carcinoma cells (124, 125). Thus, lncRNA XLOC_000647 might involve in EMT *via* NLRP3, though the molecular mechanism of the regulation in PDAC needs further definition. Similarly, P. Zhang et al.’s results showed that lncNT5E also acts as a significant modulator of its neighboring genes synaptotagmin‐binding cytoplasmic RNA‐interacting protein (SYNCRIP), which effectively regulated EMT by decreasing E‐cadherin and increasing vimentin and N‐cadherin. Nevertheless, the molecular mechanism of SYNCRIP in EMT warrants further investigation (95). In addition, Z. Li et al. found that lnc-HOTTIP contributes to EMT through upregulating HOXA13, which is located in physical continuity with HOTTIP in pancreatic cancer cells. Accompanied by EMT process, it is also revealed that HOTTIP promoted PDAC cell proliferation, invasion, and chemoresistance through regulating HOXA13. HOXA13, as a marker of gut primordial posteriorization during development, promotes lymph node metastasis and EMT, but still needs more studies of its mechanisms (96).

### LncRNAs With Unclear Mechanisms

There are still many lncRNAs play a vital part in the EMT process with unclear mechanisms that need further studies.

Jiao F. et al. reported that lncRNA metastasis-associated lung adenocarcinoma transcript-1 (MALAT-1) is associated with EMT in PDAC. They found that MALAT-1 expression increases N-cadherin, vimentin, Slug, and Snail, while it down-regulates the expression of E-cadherin. The function analysis also revealed that EMT phenotype could make further efforts to cell proliferation, migration, invasion and cancer stem-like properties *in vitro* (97). Though there was evidence indicating that MALAT1 promotes the proliferation in many solid tumors, the detailed mechanisms of MALAT-1 regulating EMT in PDAC require further exploration (126–128). Qin et al. found that lncRNA TUG1 can increase the phosphorylation of Smad2 and Smad3 as well as levels of TGF-β and TGF-β receptor protein. Thus they believed that lncRNA TUG1 promotes cell proliferation, migration, and the EMT process of pancreatic cancer cell *via* the TGF-β/Smad signaling pathway regardless of the mechanism was still unclear (74). Moreover, Dorn et al. validated the importance of LINC00261 in EMT progression, partly due to its effect on E-cadherin, and it also regulated CDH1 and TGF-β (98). Wu et al. & Zhang et al. illustrated that the overexpression of lncRNA plasmacytoma variant translocation I (PVT I) in PDAC heightens cell proliferation and EMT *via* the TGF-β/Smad pathway by downregulating p21 with an unclear molecular mechanism. Besides, p21 can also directly regulate ZEB1 and Snail expression in PDAC cells (99, 100, 103). Chen et al. substantiated that lncRNA ANRIL leads to the activation of the ATM-E2F1 signaling pathway, and E-cadherin expression decreased but N-cadherin and vimentin expressions increased, thus resulting in the promotion of EMT of PDAC cells (101). Ma et al. exposited that differential expression levels of lnc MEG3, which locates near MEG8 in human chromosome 14q32.3 region, regulates the mRNA and protein levels of Snail and EMT induction and stem cell properties in PDAC. It is speculated that Snail might be a downstream target of MEG3 that alters EMT progression (102, 129, 130). However, more studies are needed to define how Snail is regulated by MEG3 in PDAC. Y. Tang et al. expounded that the silencing of lncRNA HOX antisense intergenic RNA (HOTAIR) could prohibit the Wnt/β-catenin signaling pathway-related gene expression thereby upregulating E-cadherin, downregulating N-cadherin and inhibiting EMT. However, a more specific mechanism of how HOTAIR acts in the Wnt/β-catenin signaling pathway in PDAC is waiting to be discovered (103). C. Li et al. found that HOTAIR guided EZH2 to the promoter of miR-34a resulting in the repression of miR-34a, and the EZH2-interacting region of HOTAIR was essential in promoting cell proliferation (131). Moreover, it was approved that HOTAIR exhibits pro-oncogenic activity and acts as a negative prognostic factor in pancreatic cancer (132). Wang et al. confirmed that lncRNA-PCTST is a potential tumor suppressor in PDAC. Its overexpression increases E-cadherin and repressed vimentin expression, thus inhibiting EMT *in vitro (* 106). Similarly, Tian et al. observed that downregulation of lncRNA small ubiquitin-like modifier 1 pseudogene 3 (SUMO1P3) increases the expression of epithelial cadherin, and decreased the expression of neuronal cadherin, vimentin, and β-catenin, thus suppressing the EMT progression PDAC. But the pathway involved remains unknown (107). Moreover, Yuan et al. observed that upregulation of LINC01296 promotes the Snail-mediated process of EMT in PDAC (104). Besides, Ma et al. found increased E-cadherin accompanied by decreased N-cadherin and vimentin in lncRNA AL161431.1 knockdown PDAC cells, indicating that lncRNA AL161431.1 plays an important role in the EMT process. But the detailed mechanism of the pathway remained unknown (105).

In conclusion, lncRNAs play an important role in the EMT of PDAC. Ongoing endeavors to understand the functions of lncRNAs will help the understanding of EMT and provide opportunities to find better lncRNA-based therapeutic strategies for PDAC.

## CircRNA

**Figure 3:**
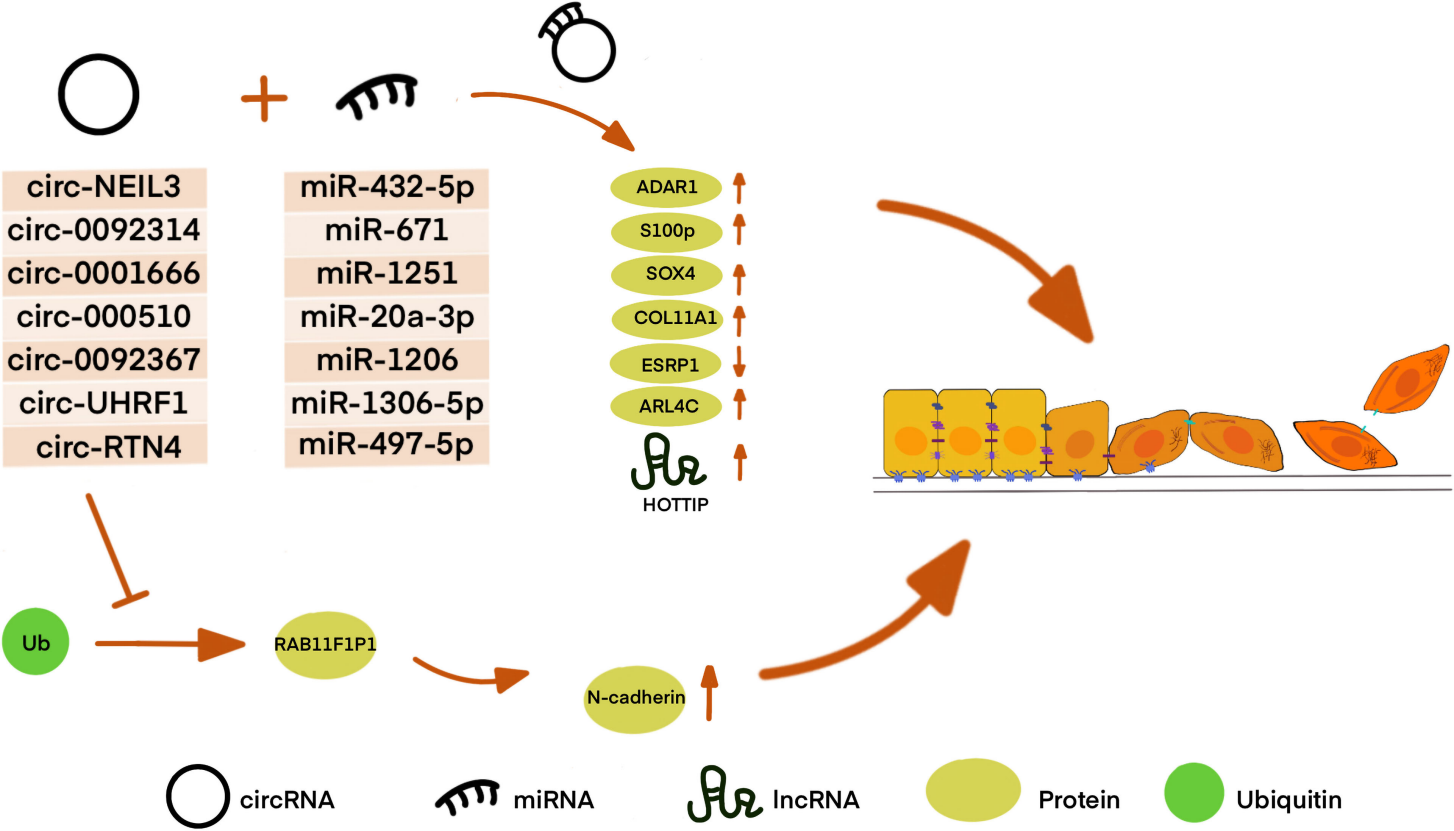
CircRNA affects EMT in many different ways in PDAC. Most CircRNAs can bind to miRNA as a sponge to regulate downstream protein or lncRNA. Circ-RTN4 is also found to regulate protein by hindering protein ubiquitination and degradation as well as acting as ceRNA.

Circular RNAs (circRNAs) are a class of ncRNA family and belong to the long-size RNAs family, which are characterized by their closed continuous loop without 5′-3′ polarity and poly(A) tail (133). Studies have demonstrated that circRNAs can involve in DNA, RNA, and also protein synthesis and gene expression (134, 135). For example, circRNAs can function as miRNA sponges or bind to other molecules to regulate mRNA expression (136, 137). CircRNA could also interact with proteins *via* dissociating bindings between proteins, cementing interactions between proteins, sequestering proteins from DNA, RNA, or other proteins. Besides, circRNAs might recruit proteins to chromatin, and then translocate or redistribute proteins (138). CircRNAs are also involved in multiple disease progression, especially in cancer (139, 140). Recently, some evidence suggests that circRNAs play an essential role in PDAC progression and EMT (**Figure 3**). The expression change in PDAC, molecular mechanisms, and related genes of these circRNAs are summarized in **Table 2**.

**Table 2:** CircRNAs in PDAC development through EMT regulation.

CircRNA	Expression change in PDAC	Relevant target genes	Known molecular mechanisms	Reference
Circ-NEIL3	Up	ADAR1	CeRNA	(141)
Circ-0092314	Up	S100p	CeRNA	(142)
Circ-0001666	Up	SOX4	CeRNA	(143)
Circ-0005105	Up	COL11A1	CeRNA	(144)
Circ-0092367	Up	ESRP1	CeRNA	(145)
Circ-UHRF1	Up	ARL4C	CeRNA	(146)
Circ-RTN4	up	HOTTIP,RAB11F1P1	CeRNA, blocking the ubiquitination site	(147)

### CircRNA Binding to miRNA as a Sponge to Inhibit miRNA Levels

For instance, P. Shen et al. demonstrated that circNEIL3 is upregulated in PDAC and is shown to regulate the expression level of ADAR1 by sponging miR-432-5p, thus inducing RNA editing of GLI1, which was previously reported to have an increased ability to activate some transcriptional targets, including Snail. ADAR1 edited the GLI1 RNA by targeting the domain in the C-terminus of GLI1, thus altering the secondary structure of the GLI1 protein. Therefore, circNEIL3 induces the EMT phenotype in PDAC cells by activating the ADAR1/GLI1/Snail signal pathway (141, 148). Moreover, Q. Shen et al. found that overexpression of circ_0092314 in PDAC tissues and cells might repress miR-671, relieving its suppression of the downstream target S100P, which acts as an EMT activator in PDAC cells by activating the AKT pathway (142). Zhang et al. investigated that circ_0001666 acts as a sponge of miR-1251, thus increasing the expression of sex-determining region Y-related (SRY) high-mobility group box 4 (SOX4) (143). SOX4 can regulate EMT by acting as a component of TGF-β signaling, enhancing the transcription of ZEB, Snail, and TWIST, and targeting growth factor receptors such as the epidermal growth factor receptor (EGFR) (149). G. Ma et al. found that circ-000510 serves as a ceRNA of miR-20a-3p and indirectly increased collagen type XI alpha 1 (COL11A1) expression, which can enhance EMT probably by the remodeling of extracellular matrix, and further impact cellular proliferation and invasion, leading to poor prognosis of patients with PDAC (144). S. Yu et al. determine that circ_0092367 regulated the expression of epithelial splicing regulator protein 1 (ESRP1) through sponging miR-1206. ESRP1, which was verified as a tumor suppressor and a prognostic factor, can also induce E-cadherin, and reduce vimentin and N-cadherin levels in PDAC. Although the mechanism remains unclear, it was identified that circ_0092367 drives EMT properties in PDAC cells by regulating the miR-1206/ESRP1 axis. In this study, they confirmed that circ_0092367 also mediates gemcitabine resistance, and its expression was positively correlated with lymph node metastasis and tumor stage and negatively associated with outcome of PDAC patients, demonstrating that circ_0092367 may be a potential prognostic biomarker for PDAC (145). Similarly, W. Liu et al. suggested that circUHRF1 directly represses the level of miR-1306-5p, thus upregulating ADP ribosylation factor like GTPase 4 C (ARL4C), which is the target of miR-1306-5p. Vimentin was downregulated in ARL4C knockdown PDAC cells, while E-cadherin was upregulated, but the mechanism was still unknown. Therefore, an in-depth investigation was required into the role of the circUHRF1/miR-1306-5p/ARL4C axis in the EMT process (146).

### More Roles of CircRNA in EMT

Although the circRNAs-miRNAs interaction is the most reported function of circRNAs in PDAC, some circRNAs are found to have additional roles.

Wong et al. identified circRTN4 interacts with RAB11FIP1 by blocking its ubiquitination site Lys578 to suppress the degradation of RAB11FIP1 and stabilize it, which can participate in the vesicle trafficking to regulate cadherin recycling. It has been provided that RAB11FIP1, which also known as Rab coupling protein (RCP), induced Slug expression through the β1 integrin/integrin linked kinase (ILK)/EGFR/Ras/NF-κB signaling pathway, thus promoting the expression of Slug, Snai1, Zeb1, Twist, and N-cadherin for EMT in PDAC. Their results also suggested that circRTN4 may function as a sponge of miR-497-5p, which can also bind with lnc-HOTTIP. Thus circRTN4 can also contribute to the EMT *via* the miR-497-5p/HOTTIP/HOXA13 pathway in PDAC (147, 150).

In brief, circRNAs play a pivotal role in the EMT of PDAC. Evidence has revealed that lots of circRNAs are changed in PDAC during the EMT process, but the functions and roles of these circRNAs are still unclear. Therefore, comprehensive studies are required to determine the functions and expression of circRNAs in EMT.

## Conclusion and Perspective

Pancreatic cancer is known to have poor prognosis, mostly due to lack of effective diagnosis at the early stage of tumor development and effective therapy. Although surgical resection of pancreatic cancers diagnosed at an early stage remains the potentially curative option for PDAC, it is only possible in a small subset of patients.

Although the functions of miRNA, a major class of ncRNA, have been extensively reported in the past decades, we are beginning to explore the implications of lncRNA and circRNA in PDAC. More and more lncRNAs and circRNAs with key functions in PDAC are discovered these years, and most of them majorly regulate a multi-step process of cancer development including EMT. LncRNAs and circRNAs are found to have oncogene as well as tumor suppressor roles and contribute to tumor progression and EMT. Mechanistically, lncRNAs can act as ceRNAs that ‘sponge’ microRNAs, bind with some EMT-related proteins, or directly regulate genes located physically nearby. CircRNAs are also known to act as ceRNAs, or regulate protein degradation. It is increasingly evident that multiple functions of lncRNAs and circRNAs are undefined. There are still challenges in the molecular mechanisms of EMT in PDAC regulated by lncRNAs and circRNAs which need an in-depth investigation. Some of those RNAs may be potential biomarkers to predict staging and metastasis of PDAC in the future. More research on that could direct new therapies and tools for liquid biopsy to patient stratification and individualized precision treatment. Understanding the regulation of lncRNA and circRNA expressions during the progression of EMT in PDAC would provide a new paradigm of clinical therapy.

## Author Contributions

WW directed and guided this study. XY and CQ collected related literature and drafted this manuscript. BZ, YW, ZL, THL, and TYL made critical revisions to this manuscript. All authors read and approved the final manuscript.

## Funding

This work was supported by the National Natural Science Foundation of China Grants (81773215, 82173074).

## Conflict of Interest

The authors declare that the research was conducted in the absence of any commercial or financial relationships that could be construed as a potential conflict of interest.

## Publisher’s Note

All claims expressed in this article are solely those of the authors and do not necessarily represent those of their affiliated organizations, or those of the publisher, the editors and the reviewers. Any product that may be evaluated in this article, or claim that may be made by its manufacturer, is not guaranteed or endorsed by the publisher.

## Abbreviations

ARL4C, ADP ribosylation factor like GTPase 4 C; AXIN2, axis inhibition protein 2; circRNA, circular RNA; COL11A1, collagen type XI alpha 1; ceRNA, competing endogenous RNA; EGFR, epidermal growth factor receptor; EMT, epithelial-mesenchymal transition;ESRP1, epithelial splicing regulator protein 1; EVs, extracellular vesicles; EZH2, enhancer of zeste homolog 2; GAS5, growth arrest-specific 5; GLI, glioma-associated oncogene; GSK-3β, glycogen synthase kinase-3β; HH, hedgehog; hnRNP I, heterogeneous nuclear ribonucleoprotein I; HIF, hypoxia-inducible factor; HIF-1α, hypoxia-inducible factor-1α; HOTAIR, HOX antisense intergenic RNA; IL-6, interleutin 6; IF, intermediate filament; JAK, Janus kinase; JNK, jun N-terminal kinase; lncRNA, long noncoding RNA; MALAT-1, metastasis-associated lung adenocarcinoma transcript-1; MAPK, mitogen-activated protein kinase; MEG8, maternally expressed gene 8; NLRP3, NOD-like receptor family pyrin domain-containing 3; ncRNA, non-coding RNA; PAX8, paired box 8; PTC, patched; PCED1B−AS1, PC−esterase domain containing 1B−antisense RNA 1; PVT I, plasmacytoma variant translocation I; RoR, regulator of reprogramming; SNHG12, small nuclear RNA host gene 12; SOCS5, suppressor of cytokine signaling 5; SOX4, sex-determining region Y-related high-mobility group box 4; SRY, sex-determining region Y-related; STAT, signal transducer and activator of transcription; SUMO1P3, small ubiquitin-like modifier 1 pseudogene 3; SYNCRIP, synaptotagmin‐binding cytoplasmic RNA‐interacting protein; TCF, T-cell factor; TGF-β, transforming growth factor-β; TME, tumor microenvironment; TNFα, tumor necrosis factor-α; TUG1, taurine upregulated gene 1; TβR1, type 1 TGF-β receptor; XIST, X-inactive specific transcript; YAP, Yes-associated protein; YB1, Y-box protein; ZEB, zinc finger E-box binding homeobox.
